# A rare homozygous variant of *CHKB* induced severe cardiomyopathy and a cardiac conduction disorder: a case report

**DOI:** 10.3389/fcvm.2024.1469237

**Published:** 2024-10-11

**Authors:** Siyuan Jing, Lu Liu, Yifei Li, Fuqiang Liu, Yimin Hua, Hongyu Duan

**Affiliations:** Key Laboratory of Birth Defects and Related Diseases of Women and Children of MOE, Department of Pediatrics, West China Second University Hospital, Sichuan University, Chengdu, Sichuan, China

**Keywords:** *CHKB*, choline, mitochondria, cardiomyopathy, neurological developmental disorder

## Abstract

**Background:**

The *CHKB* (choline kinase beta) gene plays a crucial role in regulating mitochondrial function and choline metabolism. Mutations in *CHKB* lead to conditions such as megaconial congenital muscular dystrophy (MCMD), characterized by enlarged mitochondria and impaired mitochondrial function, inducing various clinical features in neurological and cardiac performance. Herein, we report a rare case presenting with dilated cardiomyopathy as the dominant feature with a homozygous nonsense variant of *CHKB,* and the related therapeutic strategy.

**Case presentation:**

The proband, a 13-year-old male, presented with a complex clinical profile characterized by mild intellectual disability and severe cardiac impairment, including reduced activity tolerance, suspected acute heart failure, significant cardiac enlargement, a left anterior fascicular block, and a complete right bundle branch block. Whole exome sequencing (WES) identified a homozygous nonsense variant, c.598delC (p.Q200Rfs*11) of the *CHKB* gene, that resulted in disease caused by amino acid sequence changes, a truncated protein, and splice site changes, as demonstrated by MutationTaster analysis. The protein structure of CHKB was built and named AF-Q9Y259-F1. The residue around 200 amino acid sites changed in CHKB p.Q200Rfs*11 with unaltered hydrogen bonds which indicated the pathogenicity of the variant mainly originated from a truncated protein induced by the nonsense mutation. The heart blocks in the proband were considered to be associated with choline metabolic impairment, and thus cardiac resynchronization therapy would benefit the patient. Furthermore, the missense homozygous or compound heterozygous variants of *CHKB,* as well as the combined compound heterozygous missense and nonsense variants of *CHKB,* usually lead to neurological impairments and muscular weakness.

**Conclusion:**

This study expands the spectrum of *CHKB* mutations and provides essential information for the genotype–phenotype map of a nonsense variant of the gene. It is important to confirm a differential diagnosis among such patients using WES analyses. Regular cardiac and musculoskeletal monitoring is recommended for MCMD patients. Patients with a CHKB deficiency presenting with heart blocks could benefit from the administration of cardiac resynchronization therapy. This therapeutic approach might improve cardiac function and conduction in patients with CHKB-related cardiomyopathies.

## 1 Introduction

Choline, an essential nutrient, plays a critical role in various biological functions, including lipid metabolism and cellular membrane integrity, which are fundamental for cardiovascular health (1). It significantly influences cardiac function through its role in the autonomic nervous system. Acetylcholine (ACh), a neurotransmitter synthesized from choline, is pivotal in parasympathetic nervous system signaling (2). Diminished parasympathetic activity, characterized by reduced ACh signaling, has been implicated in the pathogenesis of heart failure and cardiomyopathy. Conversely, enhancing cholinergic activity has demonstrated potential benefits in improving cardiac function and mitigating pathological cardiac remodeling. The metabolism of choline involves several key enzymes, including choline kinase (CK), phospholipases, phosphocholine cytidylyltransferase (CCT), and phosphatidylethanolamine N-methyltransferase (PEMT). These enzymes orchestrate the phosphorylation of choline to form phosphocholine, which is subsequently converted into downstream substrates such as phosphatidylcholine (PC) (3–5).

The *CHKB* (choline kinase beta) gene is integral to the regulation of mitochondrial function (6). It catalyzes the initial step in phosphatidylcholine synthesis, a key component of mitochondrial membranes that is crucial for their integrity and functionality. Mutations in *CHKB* lead to conditions such as megaconial congenital muscular dystrophy (MCMD), characterized by enlarged mitochondria and impaired mitochondrial function (7). These mutations result in severe multi-complex deficiencies in the mitochondrial respiratory chain, predominantly affecting muscle and heart tissues, culminating in disorders such as cardiomyopathy. Mechanistically, *CHKB* loss-of-function mutations disrupt lipid metabolism, adversely affecting muscle fiber structure and leading to the characteristic megaconial fibers observed in muscle biopsies from affected individuals. These fibers are notably larger and more irregular compared to normal muscle fibers. Consequently, the clinical manifestations of MCMD are considered the result of impaired choline metabolism, including neonatal hypotonia, developmental delays without brain malformation, neuromuscular involvement, and myocardial dysfunction. MCMD has been reported in several publications over the past decade. Among all the reported patients, compound heterozygous variants have been predominantly identified, with missense variants being the most common. Magri et al. demonstrated differences in the clinical presentation of missense and nonsense variants of *CHKB*. In their study, heart involvement was observed in 30% of the patients, predominantly those carrying a nonsense variant of *CHKB*, which typically presented as a combination of nonsense and missense variants (7). In contrast, neurological disorders were identified in 96% of the patients, with missense variants contributing to one-third of the cases. Thus, nonsense variants of *CHKB* are more likely to induce cardiomyopathy, which is the primary cause of early death in affected individuals.

We report a rare case presenting with dilated cardiomyopathy (DCM) as the dominant feature with a homozygous nonsense variant of *CHKB* (c.598delC, p.Q200Rfs*11). This is the first documented case of a homozygous nonsense variant of *CHKB*, reinforcing the evidence of this specific variant's association with myocardial dysfunction in MCMD.

## 2 Methods

The study was approved by the ethics committee of West China Second Hospital of Sichuan University (approval number 2021-069). In addition, we obtained written informed consent from the patient’s parents prior to performing whole exome sequencing (WES) and for the inclusion of the clinical and imaging details of the patients in the publication.

The genetic test was performed on the proband and his parents. A peripheral blood sample was obtained from the patient in an ethylenediaminetetraacetic acid (EDTA) anticoagulant blood sample tube that was stored at 4℃ for less than 6 h. DNA was extracted using the Blood Genome Column Medium Extraction Kit (Tiangen Biotech, Beijing, China) according to the manufacturer's instructions. WES was performed using the NovaSeq 6000 platform (Illumina, San Diego, CA, USA), and the raw data were processed using FastP to remove adapters and filter low-quality reads. Paired-end reads were aligned to the Ensembl GRCh38/hg38 reference genome using the Burrows-Wheeler Aligner tool. Variant annotation was performed in accordance with database-sourced minor allele frequencies (MAFs) and the practical guidelines on pathogenicity issued by the American College of Medical Genetics. The annotation of MAFs was performed based on the 1000 Genomes, dbSNP, ESP, ExAC, and Chigene in-house MAF databases using the Provean, Sift, Polypen2_hdiv, and Polypen2_hvar packages in R software (R Foundation for Statistical Computing, Vienna, Austria). If there was no available full-length protein crystal structure for the targeted gene, the AlphaFold2 (https://alphafold.ebi.ac.uk/) and the AlphaFold3 (https://golgi.sandbox.google.com/) tools were used to predict the protein crystal structure and analyze the mutant site. Pymol software was then used to illustrate the molecular structures of the wild and mutant types of the targeted gene.

## 3 Case presentation

### 3.1 Clinical presentation and physical examination

The proband, a 13-year-old male adolescent, presented to our hospital's emergency department with a 3-year history of reduced activity tolerance. The patient experienced occasional fatigue and shortness of breath after walking for a few minutes. Over the previous 6 months, he had reported intermittent chest tightness and tachycardia. In the 3 days preceding admission, his condition rapidly deteriorated, with symptoms including persistent cough, lower extremity edema, orthopnea, pronounced fatigue, and suspected acute heart failure. Fortunately, no episodes of syncope or sudden cardiac arrest were reported. A detailed medical history was provided by the patient's parents. The proband had been diagnosed with cerebral palsy at 13 months of age due to significant motor retardation, likely secondary to birth asphyxia. He underwent extensive rehabilitation, which included posture correction, limb and trunk facilitation, basic movement training, assisted balance, walking, stair climbing, and running. Despite achieving average motor function milestones for his age, cognitive assessments revealed persistent developmental delays. Intellectual disability and neurological developmental impairments were confirmed using standardized scales during his school years. Notably, the patient had no history of seizures. Prior to this admission, no comprehensive cardiovascular or neuromuscular assessments had been conducted.

The proband had been delivered via cesarean section at 40 + 1 gestational weeks, with a birth weight of 3,500 g. His parents had reported no abnormalities during fetal screening. He is the second child, with an elder brother who had been deemed healthy following comprehensive cardiovascular and neurological evaluations. The family history is negative for chromosomal abnormalities, birth defects, autoimmune diseases, cardiovascular diseases, epilepsy, or neurological developmental disorders. In addition, there had been no reported exposure to teratogens during pregnancy. During a physical examination for this admission, the proband's body weight was 45 kg (25th percentile) and his height was 157 cm (25th percentile), indicating a slight developmental delay but revealed no other birth defects. His skull shape was normal, and visual and auditory evaluations were within normal limits. The patient exhibited a respiratory rate of 35–40 breaths/min, a heart rate of 110–135 beats/min, and a blood pressure of approximately 93/59 mmHg. No surface wounds were noted on his chest or other areas, indicating the absence of accidental injuries. Further physical examination showed pitting edema in both his lower extremities. His bilateral lung respiratory movements were symmetrical, with coarse breath sounds and evident wet rales. A cardiac examination revealed a regular rhythm, a dull heart sound, and significant cardiac enlargement, with the apex displaced to the left anterior axillary line. No systolic or diastolic murmurs were detected. The patient’s abdomen was soft and his liver was palpable 3 cm below the subcostal margin and 1 cm below the xiphoid process, with a medium texture; the spleen was not palpable below the subcostal margin. His muscle strength was graded at level 5 in both lower extremities and levels 4–5 in both upper extremities. Finally, the patient was negative for bilateral knee hyperreflexia and Babinski signs.

### 3.2 Laboratory and imaging evaluation

In the routine blood examination, the patient’s hepatic and renal functions were demonstrated to be in a normal range. The result of the rheumatic screening was negative. Blood tests showed elevated cTnI (0.803 μg/L; normal value <0.058 μg/L), CK-MB (9.50 μg/L; normal value <5.0 μg/L), and B-type natriuretic peptide (BNP) levels (4,870.67 pg/ml; normal value <100 pg/ml). Moreover, metabolite assessment of the patient’s blood demonstrated an increase in succinylacetone (1.52 μmol/L; normal value 0.1–1.22 μmol/L), malonylcarnitine (0.29 μmol/L; normal value 0.03–0.2 μmol/L), and butyrylcarnitine (0.52 μmol/L; normal value 0.07–0.45 μmol/L). Also, the metabolite assessment of the patient’s urine demonstrated a significant increase in hippuric acid (87.00 μmol/L; normal value 0.00–21.54 μmol/L) which has been identified as being linked with cardiovascular diseases and potential neurological impairments. Thyroid function tests indicated a normal level. Nucleic acid and autoantibody tests ruled out an infection of the Epstein–Barr, hepatitis C, or Coxsackie viruses. The cerebrospinal fluid (CSF) examination results were normal.

**Figure 1:**
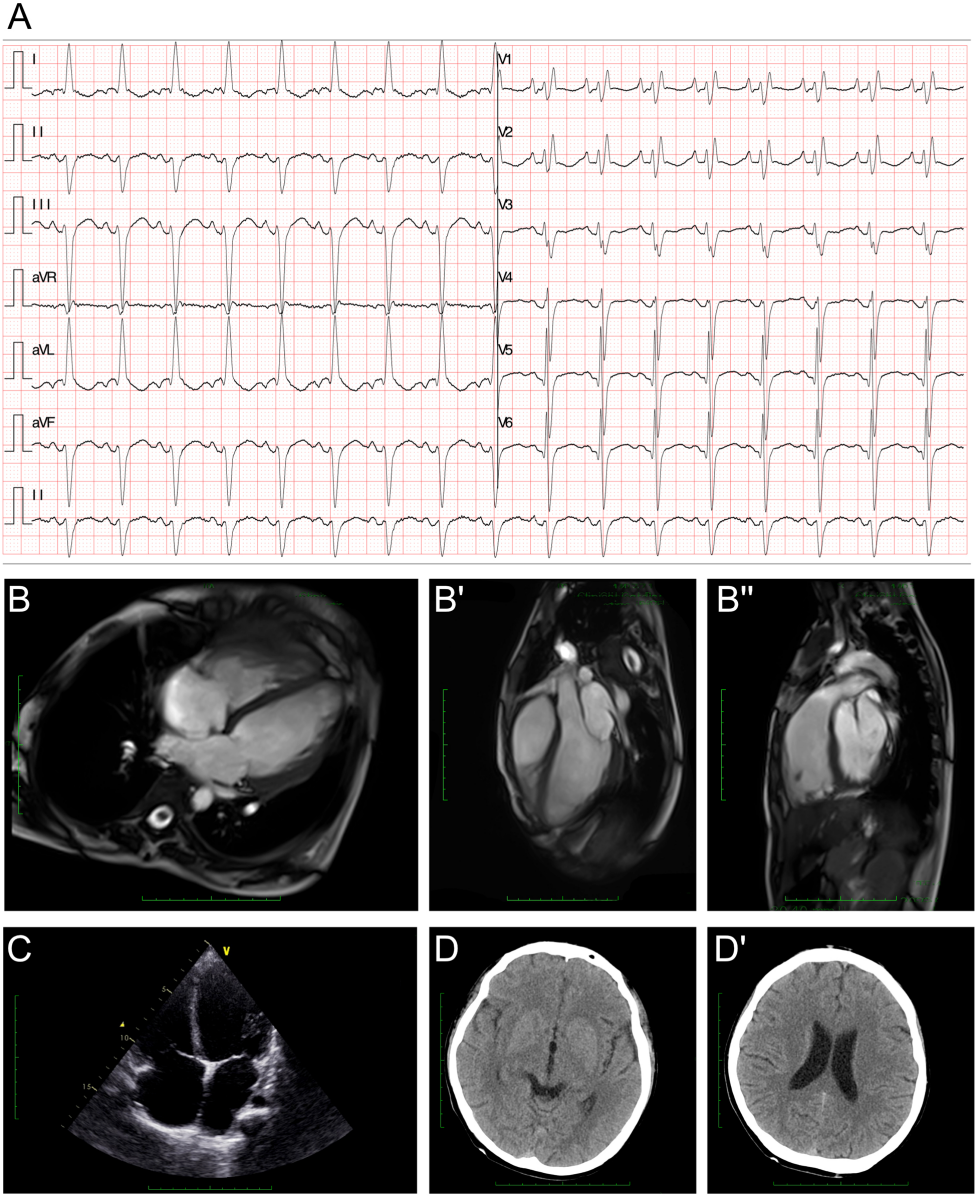
Clinical assessment of the proband. **(A)** An electrocardiogram revealed a first-degree atrioventricular block, a left anterior fascicular block, and a complete right bundle branch block. **(B–B")** A cardiac MRI demonstrated generally enlarged cardiac ventricles, a mainly affected right ventricle (RVIDd = 48 mm, LVIDd = 48 mm), severely reduced contractile function (LVEF = 14.5%, LVSV = 23.5 ml, RVEF = 6.8%, RVSV = 12.2 ml), and delayed myocardial enhancement in the left ventricle. **(C)** Echocardiography demonstrated an enlarged heart. **(D,D')** Cranial CT demonstrated normal cerebral hemisphere structures.

The electroencephalography (EEG) findings indicated a slight fully guided slow wave. The electrocardiogram (ECG) demonstrated a left anterior fascicular block and a complete right bundle branch block (Figure 1A). The cardiac MRI assessments revealed generally enlarged cardiac ventricles, a mainly affected right ventricle [right ventricular internal dimension in diastole (RVIDd) = 48 mm, left ventricular internal dimension in diastole (LVIDd) = 48 mm], severely reduced contractile function [left ventricular ejection fraction (LVEF) = 14.5%, left ventricular stroke volume (LVSV) = 23.5 ml, right ventricular ejection fraction (RVEF) = 6.8%, right ventricular stroke volume (RVSV) = 12.2 ml], and delayed myocardial enhancement in the left ventricle (Figure 1B–B"). The cardiac ultrasound indicated global heart enlargement (LV = 52 mm, RV = 35 mm), a decreased LVEF (19%, M mode method), a tricuspid annular plane systolic excursion (TAPSE) of 12 mm, and mild multi-valve insufficiency (mitral and tricuspid valves) (Figure 1C). Brain computed tomography (CT) showed normal cerebral hemisphere structures (Figure 1D and D').

### 3.3 Molecular results

**Figure 2:**
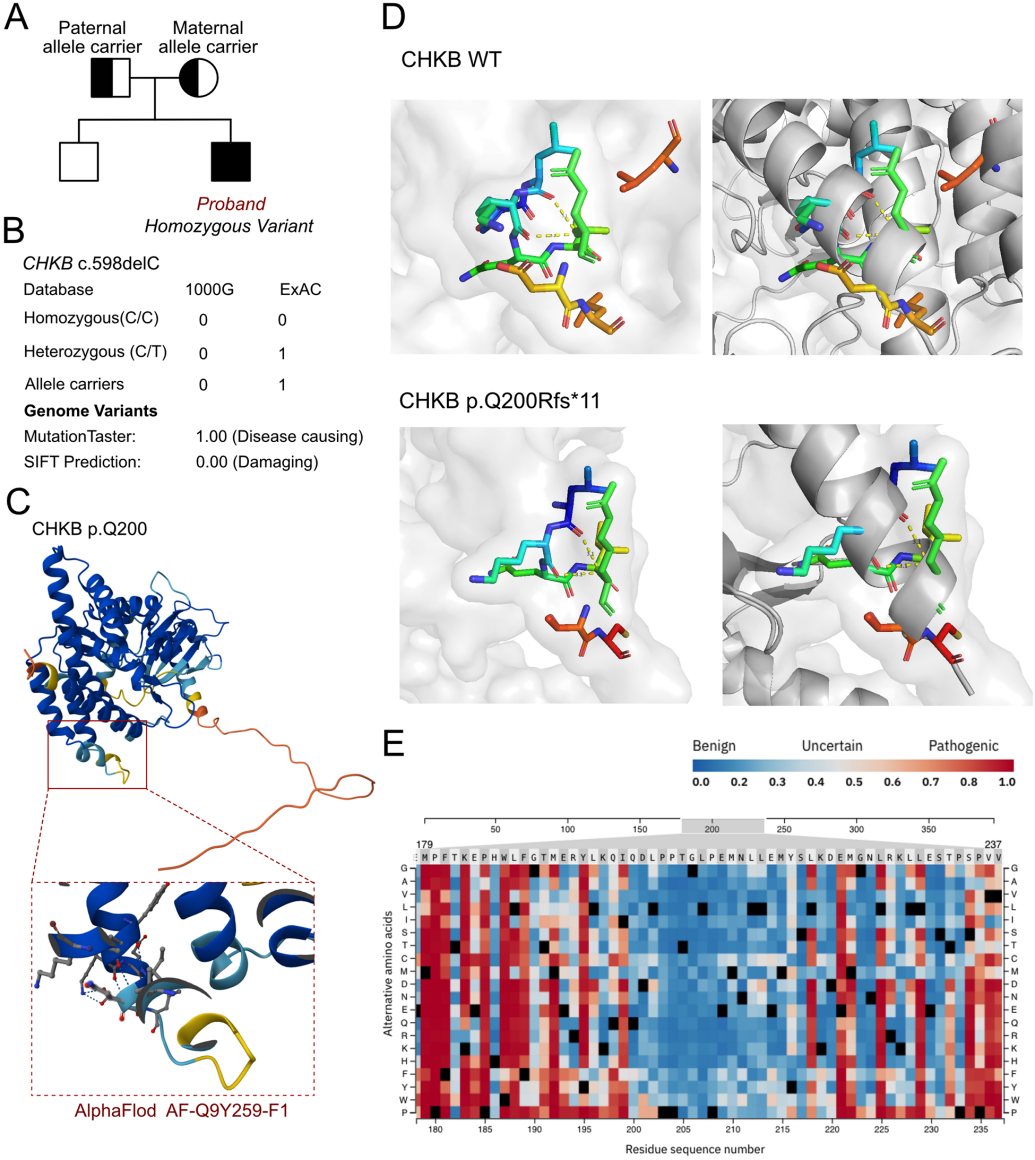
The molecular features of CHKB. **(A)** The proband exhibited a homozygous variant of *CHKB* (c.598delC, p.Q200Rfs*11). **(B)** The c.598delC variant of *CHKB* has been reported once as an allele carrier in the ExAC database and it was predicted as protein-damaging by MutationTaster. **(C)** The protein structure of CHKB was built and named AF-Q9Y259-F1. **(D)** Residue relationships around the mutant site indicated the structure changed due to the p.Q200Rfs*11 variant of CHKB, which indicated that the residue changes at the 200 amino acid sites would not significantly alter the surrounding space structure and that the pathogenicity of this variant mainly originated from a truncated protein induced by the nonsense mutation. **(E)** Pathogenicity score around the changed residue.

As cardiomyopathy was suspected, a genetic test was conducted to explore any associated variants. WES was performed for this proband and his parents. According to the WES analysis results, a homozygous variant was identified, namely, c.598delC (p.Q200Rfs*11) of the *CHKB* gene (Figure 2A). This allele variant was inherited from both the maternal and paternal sides. The c.598delC variant of *CHKB* has been reported once in the ExAC database. However, the homozygous variant has never been reported and thus was considered a novel genotype (Figure 2B). Furthermore, we excluded all the potential variants involved in cardiovascular, neurological, and muscular disorders. We then reviewed all the other variants that were reported as pathogenic or likely pathogenic, and none of these were confirmed to be associated with the phenotype of the proband, namely his intellectual disability and cardiomyopathy. Thus, we suspected the newly identified homozygous variant of *CHKB* c.598delC contributed to the pathogenic phenotype of the proband as a manifestation of a complicated clinical neurological dysfunction. To elucidate the molecular architecture of the human *CHKB* gene, we used MutationTaster with R software to predict the pathogenicity of *CHKB* c.598delC (p.Q200Rfs*11) and assessed the impact of these mutations on the protein structure. As there was no available full-length protein crystal structure for CHKB that had been analyzed by x-ray or cryo-EM, the AlphaFold2 protein structure (https://alphafold.ebi.ac.uk/) tool was used to predict the protein crystal structure. The protein structure of CHKB was built and named AF-Q9Y259-F1 (Figure 2C) (8, 9). Within the structure, the mutant site was beyond the ATP binding loop and dimer interface domain. The variant was found to induce a frameshift and lead to a large depletion of the protein, including Benner's and choline kinase motifs. We then performed a modeling analysis using the AlphaFold3 (https://golgi.sandbox.google.com/) tool for the mutant site in the wild-type with the AF-Q9Y259-F1 structure. Pymol software was then used to compare the molecular structures of the wild-type and mutant-type of CHKB. The residue around 200 amino acid sites changed in CHKB p.Q200Rfs*11 with unaltered hydrogen bonds (Figure 2D), which coincided with the pathogenic prediction at the residue of 200 amino acid sites (Figure 2E). The residue relationships around the mutant site indicated the structure had changed due to the p.Q200Rfs*11 variant of CHKB, which indicated that the residue changes at the 200 amino acid sites would not significantly alter the surrounding space structure, and the pathogenicity of this variant mainly originated from a truncated protein induced by the nonsense mutation. According to the American College of Medical Genetics, variant c.598delC has pathogenicity (PVS1 + PM2 + PM3). The MutationTaster analyses revealed that the c.598delC variant impaired the transcription of *CHKB,* leading to a frameshift, affected protein features, and amino acid sequence and splice site changes, and it was predicted to be disease-causing.

### 3.4 Final diagnosis and treatment

Upon analyzing the clinical manifestations and conducting imaging assessments and genetic tests, the patient was diagnosed with DCM and intellectual disability. However, the patient did not fully fall into the diagnostic criteria for MCMD as his muscular function remained at an average level. A long-term follow-up and therapy plan of guideline-directed medical therapy (GDMT) was developed, including the administration of vericiguat (5 mg daily), sacubitril valsartan sodium (50 mg twice a day), empagliflozin (10 mg daily), metoprolol (25 mg daily), spironolactone, and antiepileptic medication. The patient's heart function had not improved at the 3-month follow-up. Cardiac resynchronization therapy (CRT) was provided to the patient due to a prolonged QRS interval (165 ms) with a left anterior fascicular block and complete right bundle branch block.

## 4 Discussion

The *CHKB* gene, situated on chromosome 22q13.3, encodes choline kinase beta (CKβ), an enzyme integral to the biosynthesis of PC, a critical component of cell membranes (10). CKβ catalyzes the first step of the cytidine diphosphate (CDP)-choline pathway (the Kennedy pathway), converting choline to phosphocholine. This process is essential for the synthesis of PC, which maintains cellular membrane structure and function, as well as mitochondrial membrane integrity which is crucial for optimal mitochondrial performance and energy production (11, 12). Mutations in the *CHKB* gene can result in significantly reduced or absent choline kinase beta activity, leading to congenital muscular dystrophies such as MCMD (13–15). These mutations disrupt PC synthesis, impairing mitochondrial function. The resultant mitochondrial dysfunction manifests clinically as muscle weakness, the presence of enlarged muscle fibers (megaconial fibers), and cardiomyopathy (7).

As a main substrate of mitochondria, choline has been proven to participate in maintaining heart function and has potential therapeutic applications for mitochondrial disorders. Choline is essential for the synthesis of phosphatidylcholine, a major phospholipid in cell membranes, ensuring membrane integrity and proper cell signaling. Moreover, choline-derived acetylcholine influences parasympathetic nervous system activity, which can mitigate heart dysfunction by reducing sympathetic overactivity and cardiac stress. Furthermore, it modulates inflammatory responses, reducing the chronic inflammation that can lead to heart disease. Finally, choline aids in the export of triglycerides from the liver, preventing lipid accumulation and related cardiovascular issues. Thus, choline metabolism plays a curatorial role in regulating heart function. Xu et al. demonstrated that the expression of CHKB was associated with bone mineral density and body lean mass through lipid metabolism, which emphasizes the molecular function of CHKB in regulating lipid metabolism in mitochondrially enriched cells (16). Tavasoli et al. used human muscular samples and a mouse model to demonstrate that the inactivation of *CHKB* induces rostral-to-caudal muscular dystrophy (17). When changes in lipid metabolism induced by the lack of *CHKB* occur, there is an initial inability to utilize fatty acids for energy via mitochondrial β-oxidation, resulting in the shunting of fatty acids into triacylglycerol as the disease progresses. Potential therapies have been evaluated and the application of peroxisome proliferator-activated receptor agonists enables fatty acids to be used for β-oxidation and prevents triacylglycerol accumulation, while simultaneously increasing the expression of the compensatory choline kinase alpha (*CHKA*) isoform, preventing muscle cell injury (17). Thus, the total loss-of-function of *CHKB* by homozygous or compound heterozygous nonsense variants would shut down the energy production and fatty acid metabolism in myocardial or muscular tissues, which was closely associated with the pathogenic phenotype. Moreover, another research study by Tavasoli et al. further explored the molecular function of *CHKB* in maintaining heart contractile and conductive functions (6). They found that the depletion of *CHKB* induces heart failure and arrhythmic events once subjected to isoproterenol through severe mitochondrial cristae malformation and impaired electron transport chain activity (6), which could explain the left anterior fascicular block and complete right bundle branch block in the case presented in this study. Thus, CRT for this patient would improve his conduction and atrioventricular systolic compliance. This is the first report to utilize CRT techniques to reverse heart dysfunction in a patient with a CHKB defect.

In addition, transcripts derived from the *CHKB* gene have been implicated in cardiac function. Nie et al. found that long non-coding RNA *CHKB* divergent transcript (CHKB-DT) levels were significantly downregulated in the hearts of patients with DCM (18). CHKB-DT was shown to physically interact with the mRNA of *ALDH2* and fused in sarcoma (FUS) through the GGUG motif. Knockdown of CHKB-DT exacerbated *ALDH2* mRNA degradation and increased 4-HNE production, whereas overexpression of CHKB-DT reversed these molecular changes. Importantly, restoring *ALDH2* expression in CHKB-DT heterozygous depletion mice mitigated cardiac dilation and dysfunction. Furthermore, Chen et al. identified another long non-coding RNA, *CHKB* antisense RNA 1 (CHKB-AS1), which interacts with microtubule-associated protein 4 (MAP4), a key regulator in the phosphorylation of the PI3K/Akt/mTOR pathway (19). This lncRNA was demonstrated to play a role in the regulation of cellular proliferation, indicating its potential involvement in cardiac pathology.

The neurological and cardiac involvement of *CHKB* has been observed in most patients with MCMD. However, the severities of neurological and cardiac phenotypes vary among different genetic variants of *CHKB*. Mechanically, Tavasoli et al. found that *Chkb* knock-out mice failed to generate the α7β1 integrin complex, which is specific to affected muscle and neuron cells (20). In the hindlimb muscles of *Chkb* knock-out mice, there was a decrease in sarcolemma association, an abundance of the PI(4,5)-P(2) binding integrin complex proteins vinculin and α-actinin, and a decrease in actin association with the sarcolemma. The decreased vinculin localization at the plasma membrane focal adhesions was rescued by the overexpression of CHKB. Zemorshidi et al. reported 13 patients with MCDM with CHKB variants and the most common symptoms and signs included intellectual disability, delayed gross-motor developmental milestones, language skills problems, muscle weakness, autistic features, and behavioral problems (21). However, they failed to observe severe heart failure and DCM manifestation among the cases, as most of them were identified as missense compound heterozygous variants. Furthermore, Wu et al. reported two patients with CHKB variants that were initially identified as homozygous variants of SNPs (22). However, large genome deletions were further verified in the two patients, which induced giant mitochondria formation by inhibiting the mitochondrial fission process. Notably, these cases involved the nonsense variant with both small- and large-scale depletion, which should be carefully evaluated in early-onset disease or MCDM with severe heart failure. From a neurological standpoint, Pijuan et al. revealed that *CHKB* inactivation alters mitochondrial autophagy in fibroblasts, which participate in neurogenetic diseases (23). In addition, Magri et al. found reduced levels of the mitochondrial fission factor DRP1 and the severe impairment of mitochondrial respiratory chain activity in the muscle of patients with compound heterozygous variants of CHKB compared with controls (7). They demonstrated the differential diagnosis of patients with heart failure, muscular dystrophy, and intellectual disability carrying *CHKB* variants. Mitsuhashi and Nishino postulated that the decreased number of mitochondria is most likely due to increased mitochondrial clearance but it is unclear whether the muscular dystrophy is due to impaired mitochondria or excessive mitochondrial clearance (24). Vanlander et al. reported a patient who developed a rapidly progressive and life-threatening DCM and who was treated successfully by heart transplantation. They found severely enlarged mitochondria to be the most prominent feature in the skeletal and cardiac muscles. Remarkably, a subcomplex of complex V was detected, which is considered a hallmark of defective intramitochondrial protein (10). Moreover, Gong et al. also identified *CHKB* variants, namely c.1049A > G, p.H350R and c.598delC, p.Q200Rfs*11, which induced skin changes by affecting the mitochondria in keratinocytes. This was associated with dysfunction of the integrins complex, inducing clinical manifestations beyond typical neurological and cardiac involvements (25). Furthermore, two cases with the same homozygous loss-of-function variant in CHKB (c.598delC, p.Q200Rfs*11) have been reported that is different from the variant in our study. The variant led to different clinical manifestations in the two patients with normal cardiac functions (26). The explanation for this difference is unclear and may be related to the level of cytochrome coxidase (COX) deficiency, genetic modifiers, and epigenetic mechanisms.

## 5 Conclusion

In this study, we identified a homozygous variant, c.598delC, in the *CHKB* gene in the patient. Although germline mosaicism in either parent cannot be excluded, the gene-associated disease correlates well with the patient's clinical presentation. The patient exhibited severe heart dysfunction and a complex cardiac conduction disorder due to a complete loss of function of *CHKB* caused by a homozygous nonsense variant. Typically, the homozygous or compound heterozygous missense variants of *CHKB*, as well as the compound heterozygous missense and nonsense variants of *CHKB*, lead to neurological impairments and muscular weakness. Therefore, it is essential to confirm the diagnosis in these patients through WES analysis. Furthermore, choline metabolic deficiency is known to cause cardiac conduction disorders, with well-established molecular mechanisms. Patients with a *CHKB* deficiency presenting with heart blocks could benefit from CRT administration. This therapeutic approach might improve cardiac function and conduction in patients with CHKB-related cardiomyopathies.

## Data Availability

The datasets presented in this study can be found in online repositories. The names of the repository/repositories and accession number(s) can be found in the article/Supplementary Material.
